# Use of the Internet for health information by physicians for patient care in a teaching hospital in Ibadan, Nigeria

**DOI:** 10.1186/1742-5581-3-12

**Published:** 2006-12-12

**Authors:** Grace A Ajuwon

**Affiliations:** 1 Reference and Information Services Librarian, E. Latunde Odeku Medical Library, College of Medicine, University of Ibadan, Nigeria

## Abstract

**Background:**

The Internet is the world's largest network of information, communication and services. Although the Internet is widely used in medicine and has made significant impact in research, training and patient care, few studies had explored the extent to which Nigerian physicians use Internet resources for patient care. The objective of this study was to assess physicians' use of the Internet for health information for patient care.

**Method:**

172 physicians at the University College hospital (UCH) Ibadan, Nigeria; completed a 31-item, anonymous, standardized questionnaire. The Epi-Info software was used for data analysis.

**Results:**

The mean age of the respondents was 31.95 years (SD 4.94). Virtually all (98%) the respondents had used the Internet; 76% accessed it from cyber cafes. E-mail was the most commonly used Internet service (64%). Ninety percent of the respondents reported they had obtained information from the Internet for patient care; of this number, 76.2% had searched a database. The database most recently searched was MEDLINE/PubMed in 99% of cases. Only 7% of the respondents had ever searched the Cochrane Library. More than half (58.1%) perceived they had no confidence to download full-text articles from online sources such as the Health Internetwork Access to Research Initiative (HINARI). Multiple barriers to increased use of the Internet were identified including poor availability of broadband (fast connection speed) Internet access, lack of information searching skills, cost of access and information overload.

**Conclusion:**

Physicians' use of the Internet for health information for patient care was widespread but use of evidenced-based medicine resources such as Cochrane Library, Up-to-date and Clinical Evidence was minimal. Awareness and training in the use of EBM resources for patient care is needed. Introduction of EBM in the teaching curriculum will enhance the use of EBM resources by physicians for patient care.

## Background

The library and information science research community has carried out a substantial body of work examining health professionals' information needs, information seeking and use [1-5], and [6]. Among health professionals, physicians represent the majority of populations studied [7]. Physicians seek health information for various reasons: the need to obtain answers to patient-specific questions [8] and to keep abreast of developments in clinical medicine [8,9]. Thompson [8], in her study of the characteristics of information resources preferred by primary care physicians found that above all, physicians seek information for patient care. Traditionally, printed materials such as books, journals held in personal libraries and colleagues were physicians' main sources of information [8,10,11], and [12]. However, with the increase in the pace of health care research and the introduction of computers and the Internet, many new electronic information resources and systems are now available [13]. The availability of computers and especially the Internet has provided the possibility of immediate access to the most recent and reliable results of clinical research in everyday medical practice in developed countries [14]. In developing countries on the other hand, the Internet is still only available to a minority of health professionals, and often it is not available at the point of care.

Evidence-based medicine (EBM) is about solving clinical problems. It is an approach to clinical decision making that emphasizes data instead of opinion [15]. EBM relies on identifying and reviewing the best and most relevant scientific literature to determine the value of diagnosis, test or treatment; because of this, EBM provides a powerful tool for better patient care [15]. Unfortunately, research consistently has shown that clinical decisions rarely are based on the best available evidence [16]. EBM differs from traditional medical practice because it acknowledges that intuition, unsystematic clinical experience, and path-physiologic rationale are insufficient grounds for clinical decision making and it stresses the examination of evidence from clinical research [17].

Several studies have explored the use of the Internet to obtain clinical information for patient care by medical practitioners in diverse health institutions across the globe. For example, a 2004 study of resident physicians' adoption of information technology in Pennsylvania, United States, showed that 98% of the respondents used the Internet and two-thirds used it for health related purposes [18]. Similar studies from the United States also reported that physicians sought information from the Internet for patient care [19,20]. Findings from New Zealand studies showed that a greater proportion of General Practitioners (GPs) and Family Physicians (FPs) have access to the Internet and the majority used it for patient care [21,22]. A similar finding was also reported by Veness among Australian and New Zealand radiation oncologist and registrars; 85% of the respondents considered medical research findings 'useful' in day-to-day management of patients [23].

The Nigerian health care sector is divided into three namely: Primary Health Care (PHC), Secondary and Tertiary services. Primary Health Care (PHC) is closest to the people and is constitutionally the responsibility of Local Governments (LGs). PHC services are available in rural and semi-urban centers in the country. In some rural communities, the PHC centers lack qualified medical practitioners and nurses provide services. Difficult cases that are beyond the health workers in PHC centers are referred to the nearest Secondary Health Care services in the area.

Secondary Health Care services are meant to take care of health problems that cannot be solved at the PHC level. Services at the Secondary Care are delivered in General or District hospitals under the supervision of the State governments. The General and District hospitals are located in towns and semi-urban centers with qualified medical practitioners that attend to patient's health problems. Some of the General and District hospitals do not have specialists to take care of rare and very difficult cases as a result; patients are referred to specialist hospitals located in capital cities.

The Tertiary Health Care service, which is the most sophisticated and costly for government and patients, are located in capital cities. These include University Teaching Hospitals and Federal Medical Centers as well as the National Hospital in Abuja. The Tertiary Health Care deals with the most difficult cases referred from Secondary Health Care services. There are many specialists in different fields of medicine at the tertiary health care level. All the university teaching hospitals in the country are also training institutions and are supervised by the Federal Ministry of Health [24].

Both the primary and secondary health care services are available in rural communities and towns with limited or no access to Internet facilities. The tertiary health care are located in big cities where there is Internet connection however, many of the health workers do no have connection in their offices, in cases where there is connection to the Internet, epileptic power (electricity) supply is a major challenge.

Internet access is still a major challenge in Nigeria because the majority of people cannot afford the high initial cost of personal computers and connection fees. This has compelled most Nigerians to access the Internet via cyber cafes. Access to the Internet at home is not a common phenomenon as it is with cell phones. Cyber cafes are popular in Nigeria because access to the Internet is fee-based irrespective of where the service is used. Unfortunately, use of cyber cafes has several constraints including high cost of access, lack of privacy, and the fact that these cafes are typically rowdy. Other problems include the fact that several cafés do not install necessary software (e.g. Adobe Acrobat Reader) on their computers which makes it difficult for users to download or open documents/articles in Portable Document File (PDF) format. In some cafes, the computers do not have USB ports; some cafes have only few computers with functional floppy disk drives. As a result, downloading from the Internet into USB storage device (flash disc) and or floppy disk becomes impossible.

While previous studies cited above showed use of the Internet for clinical information for patient care by practitioners in developed countries, little is known about the extent to which their counterparts in developing countries use the Internet for patient care. This study was designed to fill this gap. The objective of the study was to determine the extent to which physicians' at the University College Hospital (UCH), Ibadan, Nigeria use the Internet to obtain health information for patient care.

## Methods

### The setting

The UCH, Ibadan, was established in 1957 and is the oldest federal institution for tertiary health care, teaching and research in Nigeria. The hospital has 45 medical specialties and subspecialties, runs 75 weekly consultative clinics and has 805 beds. Apart from providing care, UCH is also a major training institution for all categories of health workers in the country. Through the College of Medicine, University of Ibadan, UCH has trained over 5, 000 physicians and dentists and has produced approximately an equal number of scholarly publications of health-related research. The hospital itself has trained more than 6, 000 nurses and midwives since inception and several hundred other health professionals including medical laboratory scientists, teachers of community health, environmental health, medical records and radiography. To date over 12 million patients have received care in the hospital [25].

### Study population

The study population consisted of House Officers (HOs), Registrars (REGs) in residency training and Medical Officers (MOs) at the Staff Medical Services (Staff Clinic) and General Outpatient Department (GOPD) in UCH. The Staff Medical Services run daily consultative clinics for the staff of UCH and their family members and those of the College of Medicine and their families. The Staff Medical Services is a fully-fledged department in UCH and the patients consult MOs and specialists in General Practice (GP). All three categories of respondents (REG, MOs and HOs) were working in UCH between October and November 2005 when the study was conducted.

### Measures

A 31-item anonymous questionnaire was designed and used to collect data for the study (See additional file 1). It was developed from a review of the literature and two previous studies conducted by the author among medical students and student nurses [26] as well as medical and dental students [27] in the same institution (UCH). The questionnaire elicited information about demographic profile, computer literacy, use of health information from search engines and databases for patient care, perceived confidence in performing Internet related tasks and problems encountered in using the Internet. It consisted of two open-ended questions and twenty-nine close-ended questions for ease of administration. The face validity of the questionnaire was assessed by given it to library colleagues for perusal. It was also pre-tested among 12 randomly selected REGs and HOs from the Faculty of Dentistry. As a result, HOs and Registrars in that Faculty were excluded from the study.

### Recruitment and method of data collection

As part of preparation for the study, the investigator enumerated the physicians in residency (REGs), those on internship (HOs) and MOs by reviewing records kept at the Establishment/Personnel office in UCH. This revealed about 500 physicians available for study. The author obtained official permission to administer the questionnaire from Heads of Departments and Chief Registrars. The initial plan was to enroll all eligible physicians into the study. However, preliminary investigation showed that weekly meetings including Grand Rounds and Seminar Sessions were suitable avenues for reaching many of the REGs and HOs. Consequently, the dates and times of the meetings were recorded and the author attended the scheduled meetings between October and November 2005. During these meetings a total of 250 questionnaires were distributed to all eligible respondents (HOs and REGs) in attendance. It was administered to Registrars and HOs shortly before the meeting commenced and was collected after it was concluded. For MOs, the questionnaire was distributed to them through their Departmental office and completed questionnaires were kept there for the investigator to collect. Informed consent was given by return of completed questionnaire. The remaining 250 physicians were either on vacation or absent from the meetings as a result of busy schedules during the study period. Also, some of the physicians were on clinical rotation in other departments that did not grant the investigator permission to administer questionnaires to eligible respondents in their department.

### Data analysis

The questionnaires were reviewed for completeness; they were collated and numbered serially. Open-ended questions were coded and were entered into the computer. Analysis was descriptive. The EPI-Info software developed by the Centre for Disease Control in Atlanta, Georgia, U.S.A. was used for data analysis.

## Results

Of the 250 questionnaires distributed, 172 were completed and returned (response rate 69%).

### Demographic profile of respondents

The demographic profile of the respondents is described in Table 1. The ages of the respondents ranged from 25 to 50 years with a mean of 31.9 (SD: 4.94). There were more males (67.4%) than females (32.6%). Registrars constituted the majority (60.5%). The affiliation of the respondents showed that the majority (79.1%) were from the Faculty of Clinical Sciences (FCS).

**Table 1:** Demographic profile of physicians in a teaching hospital in Ibadan, Nigeria (N = 172)

Variables	No	%
Age (in years)		
25–30	68	39.5
31–36	83	48.3
37–42	16	9.3
43 and above	5	2.9

Gender		
Male	116	67.4
Female	56	32.6

Current Status/Position		
House officers	45	26.2
Medical Officers	23	13.4
Registrars	104	60.4

Faculty		
Clinical Sciences	136	79.1
Basic Medical Sciences	27	15.7
Public Health	9	5.2

Total	172	100

### Computer literacy and Internet usage

Respondents' computer literacy levels and Internet usage are presented in Table 2. Overall, 93% of the respondents were computer literate while 7% were not. Of the 7% that were not computer literate, 33.3% said they did not have the time to learn it, 33.3% said they had no access to a computer, 8.4% had no interest and the remaining 25% did not answer the question. More than half (58%) of the respondents reported that they owned a personal computer (PC). The proportions of computer owners by status showed that more REGs (69.2%) own a PC than MOs (56.5%) and HOs (31.1%). Virtually all the respondents (98%) had used the Internet; of this number, 67.3% claimed they used it during the week preceding the study.

**Table 2:** Computer literacy and Internet usage among physician's in Ibadan, Nigeria

	House Officers (N = 45)		Medical Officers (N = 23)		Registrar (N = 104)		Total (N = 172)	
	No	%	No	%	No	%	No	%

Are you computer literate?								
Yes	41	91.1	22	95.7	97	93.3	160	93.0
No	4	8.9	1	4.3	7	6.7	12	7.0

Do you own a personal computer?								
Yes	14	31.1	13	56.5	72	69.2	99	57.6
No	31	68.9	10	43.5	32	30.8	73	42.4

Ever used the Internet?								
Yes	44	97.8	22	95.7	102	98.1	168	97.7
No	1	2.2	1	4.3	2	1.9	4	2.3

Used the Internet during the last week preceding the survey?								
Yes	19	42.2	14	60.9	80	76.9	113	65.7
No	25	55.6	8	34.8	22	21.1	55	32.0
No Response	1	2.2	1	4.3	2	1.0	4	2.3

Do you have personal access to the Internet?								
Yes	7	15.6	12	52.2	43	41.3	62	36.0
No	38	84.4	11	47.8	61	58.7	110	64.0

Main reason for using the Internet the last time.								
E-mail	34	75.6	11	47.8	62	59.6	107	62.2
Research	2	4.4	3	13.0	16	15.4	21	12.2
Health information	3	6.7	5	21.7	16	15.4	24	14.0
Patient care	2	4.4	1	4.4	6	5.7	9	5.2
Others	1	2.2	1	4.4	1	1.0	3	1.7
No response	3	6.7	2	8.7	3	2.9	8	4.7

Internet access points used the last time.								
Office	1	2.2	5	21.7	14	13.5	20	11.6
Home	2	4.5	0	0.0	1	1.0	3	1.7
Library	3	6.7	1	4.4	14	13.5	18	10.6
Cyber café	3 8	86.4	16	69.5	73	70.1	127	73.8
No Response	1	2.2	1	4.4	2	1.9	4	2.3

Mode of Internet usage								
Assisted	7	15.6	3	13.0	18	17.3	28	16.3
Not Assisted	37	82.2	19	82.6	84	80.8	140	81.4
No Response	1	2.2	1	4.4	2	1.9	4	2.3

Total	45	100	23	100	104	100	172	100

Respondents' were asked if they had personal access to the Internet; approximately one-third (36%) reported having Internet access while two-thirds (64%) had no access. Of the 36% who had personal access to the Internet, 52.2% were MOs. Respondents were asked which outlet they used in accessing the Internet the last time; a majority (76%) said they did so from cyber cafes. The majority (83.3%) claimed they used the Internet unassisted while someone assisted 17%. Respondents were also asked the main reason they used the Internet the last time; communication by email (65.2%) tops the list of reasons cited.

### Use of health information and search engines

Respondents' use of health information on the Internet and search engines is presented in Table 3. Overall, 90% of the respondents claimed they had obtained health information from the Internet for patient care. More REGs (94.1%) used the Internet to retrieve information for patient care than MOs (81.8%) and HOs (84.1%). The respondents were asked which search engines they used to obtain information from the Internet. Google and Yahoo were the popular search engines reported by the respondents; about two-thirds (63%) searched Google and (36.1%) visited Yahoo. Overall, 70% had used a search engine during the month preceding this study. More REGs (78.4%) used it than HOs (56.8%) and MOs (54.5%).

**Table 3:** Use of health information and search engines by physicians in Ibadan, Nigeria

	House Officers (N = 45)		Medical Officers (N = 23)		Registrars (N = 104)		Total (N = 172)	
	No	%	No	%	No	%	No	%

Ever obtained health information from the Internet for patient care?								
Yes	37	82.2	18	78.3	96	92.3	151	87.8
No	7	15.6	4	17.4	6	5.8	17	9.9
No Response	1	2.2	1	4.3	2	1.9	4	2.3

Search Engine used to obtain health information								
Yahoo	17	37.8	6	26.1	34	32.7	57	33.1
Google	23	51.1	13	56.5	63	60.6	99	57.6
Others	0	0.0	0	0.0	2	1.9	2	1.2
No Response	5	11.1	4	17.4	5	4.8	14	8.1

Used a Search Engine during the previous month?								
Yes	25	55.6	12	52.2	80	76.9	117	68.0
No	19	42.2	10	43.5	22	21.2	51	29.7
No Response	1	2.2	1	4.3	2	1.9	4	2.3

Total	45	100	23	100	104	100	172	100

### Use of databases for health information

Respondents' use of databases as sources of health information is presented in Table 4. Respondents were asked if they had ever searched a database before; 76.2% said they had done so while 23.8% had not. Respondents were asked which of the databases they searched on the Internet the last time, 99.2% reported they searched MEDLINE/PubMed and 0.8% Cochrane. Two-thirds (64.5%) of the physicians claimed that they obtained relevant information from the database for patient care. When asked which of the Evidence-Based Medicine (EBM) resources they had used, about two-thirds (61%) of the respondents did not answer the question. Of those who did, only 7% had used the Cochrane Library and 16% used Clinical Evidence.

**Table 4:** Use of Databases for health information by physicians in Ibadan, Nigeria

Variable	House officers (N = 45)		Medical Officers (N = 23)		Registrars (N = 104)		Total (N = 172)	
	No	%	No	%	No	%	No	%

Ever searched a database								
Yes	32	71.1	16	69.6	83	79.8	131	76.2
No	13	28.9	7	30.4	21	20.2	41	23.8

Which of these databases did you search the last time?								
MEDLINE/PUBMED	32	71.1	16	69.6	82	78.8	130	75.6
Cochrane	0	0.0	0	0.0	1	1.0	1	0.6
No Response	13	28.9	7	30.4	21	20.2	41	23.8

Did you obtain relevant health information from the database for patient care?								
Yes	25	55.6	15	65.2	71	68.3	111	64.5
No	20	44.4	8	34.8	33	31.7	61	35.5

Online Evidence Based Medicine sources ever searched.								
Cochrane	2	4.4	2	8.7	8	7.7	12	7.0
Up-to-date	1	2.2	0	0.0	8	7.7	9	5.3
Clinical Evidence	7	15.7	7	30.4	13	12.5	27	15.7
Others	2	4.4	0	0.0	17	16.3	19	11.0
No Response	33	73.3	14	60.9	58	55.8	105	61.0

Total	45	100	23	100	104	100	172	100

### Perceived confidence to perform Internet related tasks and problems encountered

The respondents' perceived confidence in performing six Internet related tasks is shown in Table 5. Approximately 80% were not confident to download textbooks from the Internet; 45% were confident to obtain information to perform a clinical procedure. More REGs 50% than HOs (42.2%) and MOs (25.1%) perceived they could perform this task. Sixty-six percent reported they had the confidence to search the Internet for information on diagnosis and treatment. More than half 58.1% perceived they had no confidence to download full-text articles from online sources such as the Health Internetwork Access to Research Initiative (HINARI).

**Table 5:** Perceived confidence of physicians to perform Internet related tasks

Tasks	House Officers (N = 45)		Medical Officers (N = 23)		Registrars (N = 104)		Total (N = 172)	
	No	%	No	%	No	%	No	%

Retrieve and download free medical books from the Internet								
Yes	4	8.9	5	21.7	26	25.0	35	20.3
No	41	91.1	18	78.3	78	75.0	137	79.7

Search the Internet to find out how a particular clinical procedure is carried out								
Yes	19	42.2	5	26.1	52	50.0	77	44.8
No	26	57.8	17	73.9	52	50.0	95	55.2

Search the Internet for the most current diagnostic test or therapy for a disease condition								
Yes	26	57.8	18	78.3	69	66.3	113	65.7
No	19	42.2	5	21.7	35	33.9	59	34.3

Retrieve and download full-text articles from online journals, BJM; HINARI etc.								
Yes	8	17.8	12	52.2	52	50.0	72	41.9
No	37	82.2	11	47.8	52	50.0	100	58.1

Find the most current available evidence to answer a clinical question relating to patients condition								
Yes	24	53.3	12	52.2	66	63.5	102	59.3
No	21	46.7	11	47.8	38	36.5	70	40.7

Find information on the diagnosis, prognosis and treatment of an ailment								
Yes	39	86.7	20	87.0	93	89.4	152	88.4
No	6	13.3	3	13.0	11	10.6	20	11.6

Total	45	100	23	100	104	100	172	100

The problems respondents encountered while using the Internet are presented in Table 6. About two-thirds (62%) of the respondent's encountered problems searching the Internet while 38.4% did not. Of all the problems listed, slow Internet connection was a problem faced by 44% of the respondents, followed by lack of information searching skills (26%). Other challenges were information overload and lack of skills to efficiently obtain needed information.

**Table 6:** Problems encountered searching the Internet by physicians' current status

	House officers (N = 45)		Medical officers (N = 23)		Registrars (N = 104)		Total (N = 172)	
	No	(%)	No	(%)	No	(%)	No	(%)

Did you encounter any problem using the Internet?								
Yes	27	60.0	14	60.9	65	62.5	106	61.6
No	18	40.0	9	39.1	39	37.5	66	38.4
Problems encountered								
Slow Internet connection	20	44.4	9	39.1	46	44.2	75	43.6
Too much information	13	28.9	3	13.1	9	8.6	25	14.5
Cost of Internet Access	0	0.0	4	17.4	24	23.1	28	16.3
Lack information searching skills	12	26.7	7	30.4	25	24.1	44	25.6
Total	45	100	23	100	104	100	172	100

The respondent's in this study namely: HOs (27%), MOs 30.4% and REGs 24.1% claimed they were overwhelmed by the amount of health information on the Internet. Cost of Internet access was also mentioned by 16.3% as one of the challenges they face in using the Internet. While cost of access was not so much of a problem for HOs (0.0%), it was for MOs (17%) and REGs (23.1%).

## Discussion

The Internet as a medium of communication is useful in medicine; and has become an important means of how physicians' deliver care. Virtually all the respondents in this study 98% had used the Internet. This figure is comparable to 98% resident physicians in University of Pennsylvania Medical School [18] and 96% among Medical and Dental students in UCH [27]. This finding is evidence of how ubiquitous the Internet has become even in tertiary institutions in a developing country; although Internet access is much less available at primary and district levels. The Internet is a fast means of communication and contains more varied information than one could ever imagine. Consequently anyone who has access and the required skills will be able to obtain relevant information from the maze of available information on the Internet.

The majority of the respondents in this study relied on the Internet for information because it has a lot to offer. Due to funding constraints, paper-based libraries in Nigeria are no longer able to meet the needs of users as new books and current journals are few and in some cases not available at all. Also, most of the libraries in Nigeria, particularly health science libraries, have few if any subscriptions to online journals and databases. As a result, users depend on the free resources that are readily available on the Internet, some of which are meant for people in developing countries. The availability of these free information sources may explain why virtually all the respondents in this study used the Internet as a source of health information.

In this study, few (36%) physicians have their own private Internet connection and the majority does not have access at work. The UCH authorities have not provided this service to staff in their offices or consulting rooms due to funding constraints. Lack of access to the Internet in the office may undermine physicians' ready access to up-to-date information for patient care. The availability of computer laboratories in a few departments may explain why 12% of the respondents said they have access at work. It is not surprising therefore that the majority (76%) of the respondents used the cyber cafes as the setting to access the Internet.

E-mail was the most commonly used Internet service and was the main reason why two-thirds (65.3%) of the respondents in this study used the Internet. This figure is comparable to studies among physicians in rural Washington 60% [28] and Vienna, Austria 60% [8]. However, it is lower than 96% among resident physicians in Pennsylvania, United States [18]. E-mail is a fast and reliable means of communication compared to surface mail/postal services in Nigeria. This is due to the slow pace of postal services in the country. The high cost of sending documents through facsimile has further popularized e-mail. Since individuals can scan their documents and send it as attachment via e-mail, more people tend to use this method, including physicians.

Ninety percent of respondents claimed they had obtained information from the Internet for patient care. This figure is higher than 7% among medical doctors in Switzerland; 49% family physicians in New Zealand, 58% primary care practitioners and 60% resident physicians in the United States [9,21,20] and [18]. Due to high cost of books and subscription-based journals, only few individuals can afford to subscribe to journals while the majority relies on the medical library for needed information. Unfortunately, health sciences libraries in Nigeria have not been able to meet the needs of most of its users due to shortage of funds. This development has resulted in health professionals relying more on the Internet for health information. Also, the Open Access initiative has made it possible for articles published in some electronic journals to be accessed freely on the Internet. HINARI [29] and other digital archives have made it possible for researchers and health professionals in developing countries to have free access to full-text articles of some electronic journals and databases. This is also true of the Program for the Enhancement of Research Information (PERI) [30] of the International Network for the Availability of Scientific Publications (INASP). The availability of these free resources may explain why a high percentage of the respondents used the Internet to obtain health information relating to patient care.

MEDLINE/PubMed was the most frequently accessed database by virtually all the respondents (99.2%). This figure is higher than (38%) in Switzerland [31] and 40% in Washington State [32]; 74% among Australian and New Zealand radiation oncologists [23] and 70% among New Zealand family practitioners [21]. The finding of this study regarding use of MEDLINE differs from a previous study in the United States where 40% Primary Health Practitioners never perform a literature search from online sources such as MEDLINE [20]. Also, MEDLINE has an impressive coverage of biomedical research findings with abstracts and in some cases free full-text articles from PubMed Central, Biomed Central, Bioline International, Directory of Open Access Journals (DOAJ) and other digital archives. Many of those who search MEDLINE/PubMed were confident of getting relevant health information that will serve their needs. This may explain why many of the respondents search this database. Another important reason MEDLINE/PUBMED was used instead of other databases is that most of the other databases such as EMBASE require a subscription before they can be accessed; PubMed is popular because it is free on the Internet. Also, the availability of online books through PubMed may explain why many of the respondents rely on this database for needed health information. The database has a broad coverage of the biomedical and allied health literature making it an attractive option for many health professionals, including the respondents in this study. However, individual research articles can be very misleading hence the importance of systematic reviews/EBM resources.

The majority (61%) of the respondents in this study did not answer the question on the use of EBM resources for patient care. Possibly many of them are not aware of the existence of these resources. The few who are aware may not know how to use them for patient care purposes. Another possible explanation could be the fact that many of the respondents still prefer interpersonal sources such as their colleagues and printed materials (books and journals) as information sources in order to solve clinical problems. The low utilization of EBM resources by the respondents in this study could also be due to the fact that there are few advocates of EBM in the country and the practice of EBM is not yet a part of the teaching curriculum. Previous studies have shown that though clinicians may be aware of EBM, few use it in clinical practice [33]. A recent survey of 190 obstetricians and family physicians in obstetric practice in the Canadian province of Saskatchewan found that although 76% of the respondents said they were aware of EBM most did not use it to solve difficult clinical problems. Instead, 51% consulted a respected authority, 37% consulted a textbook, and only 8% conducted a Medline search [15]. Also, a 2005 study of evidence-based reproductive health care among health workers in Cameroon revealed that only 25% of the respondents had access to a library or the Internet or email. Also, awareness of the evidence-based World Health Organization (WHO) Reproductive Health Library was low [34]. Young's study of GPs use of evidence databases in New South Wales showed that 22% of the respondents were aware of the Cochrane Library, only 6% had access to it and 4% had ever used it [35].

## Conclusion

Internet usage is widespread among physicians, however, use of online EBM resources such as the Cochrane Library, Clinical Evidence and Up-to-date was very minimal. Awareness and training in the use of EBM resources for patient care is needed. Introduction of EBM into the existing curriculum would enhance the ability of physicians to acquire, appraise and use EBM resources to solve patient's health problems.

## Conflict of interests

The author(s) declare that they have no competing interests.

## Supplementary Material

Additional file 1 Internet use by Resident doctors, Medical and House Officers for patient care. The document provided is a 31-item questionnaire used for data collection. Click here for file
