# Supplementary material for: Use of the Internet for health information by physicians for patient care in a teaching hospital in Ibadan, Nigeria
Source: Biomed Digit Libr. 2006 Dec 12;3:12. doi: 10.1186/1742-5581-3-12 (PMC1764751; doi:10.1186/1742-5581-3-12)
Supplement: Additional file 1 — Internet use by Resident doctors, Medical and House Officers for patient care. The document provided is a 31-item questionnaire used for data collection. [file 1742-5581-3-12-S1.doc]

Internet use by resident doctors medical and House officers for patient care

The purpose of this interview is to collect information on the use of Internet for patient care by Resident doctors and Medical Officers in University College Hospital (UCH), Ibadan. The information obtained from this questionnaire will be used to for research purposes only. Please note that your participation in this study is voluntary.

Thank you for consenting to be a participant in this study.

Instruction: Please read the following questions carefully and circle the appropriate one.

Section A: Demographic characteristics of the respondents

1. Age: 1. 25-30 2. 30-35 3. 35-40 4. 40 and above
2. Sex: 1. Male 2. Female
3. Current status: 1. House Officer 2. Medical Officer 3. Registrar
4. Marital status: 1. Single 2. Married
5. Department: ----------------------------------------------
6. Specialty: --------------------------------------------------

# Section B:

# Instruction: Please circle the option that applies to you.

7. Are you computer literate? 1. Yes 2. No (**if NO, go to Q8**)

8.Why are you not computer literate? 1. I don’t have the time to learn it

2. I have no access to a computer system 3. I’m not interested

4. It is not useful to me 5. Others, specify ---------------------------

9. Have you ever used the Internet? 1. Yes 2. No (**if NO, go to Q17)**

10. Have you used the Internet during the last one-week?

1. Yes 2. No

11. What was your main reason for using the Internet the last time? **(Please choose one)**

1. E-mail 2. Research 3. Health information

4. Patient care information 5. Business 6. Others (specify) --------------

12. Have you ever browse the Internet in order to obtain health information relating to

patient care? 1. Yes 2. No

13. What specific health information did you obtain from the Internet the last time you

did? 1. Aetiology of health condition 2. Diagnosis 3. Treatment 4. Prognosis 5. Others ----------------- (please specify).

14. Which search engines did you use to obtain this information? 1.Yahoo

2. Google 3. Alta Vista 4. Ask Jeeves 5. SCIRUS 6. Others, specify ----

15. Have you done so during the last one-month? 1.Yes 2. No

16. Did you search the Internet yourself or someone assisted you the last time?

1. Assisted 2. Not assisted

17. Where did you use Internet service the last time? 1. Office 2. Home

3. Library 4. Cyber café 5. Others, specify -----------------------

18. Have you ever searched a database before? 1. Yes 2. No (if No, go to Q19)

19. Which database did you search the last time? **(Please, choose one**)

1. MEDLINE / PUBMED 2. EMBASE 3. COCHRANE 4. POPLINE 5. Others, please specify ---------------------

20. Did you obtain relevant health information for patient care from the database?

1. Yes 2. No

21. Did you encounter any problem in searching for information on the Internet?

1. Yes 2. No

22. What major problem did you encounter? **(Please, choose one)**

1. Slow Internet connection 2.Too much information 3. Cost of Internet access

4. Don’t know were to find information 5. Lack information searching skills

6. Others, specify-------------------------------

23. Which of the following online medical information sources have you ever searched

for the best clinical evidence for patient care? **(Please, choose one)**

1. ACP Journal Club
2. Best Evidence
3. Cochrane
4. Up-To-Date
5. Clinical Evidence
6. Clinical Queries
7. Others, specify ------------------------------------------------

23. Do you have a personal computer? 1.Yes 2. No (If Yes,

24. Do you have access to the Internet either at home or at work? 1.Yes 2. No

25. Which of the following areas would you require training (**please chose one)**

1. Literature searching: Pub Med, Cochran
2. Internet sites for medical information
3. Accessing free full-text electronic journals through HINARI
4. Downloading text books and other resources
5. Sources for best clinical evidence for patient care

Section C: Skills and Confidence in using Internet services (tick the appropriate number)

| Have you ever performed any of the following tasks? | Yes  1 | No  2 | Have you done so during the last six months?  Yes No  1 2 | |
| --- | --- | --- | --- | --- |
| 26. Downloaded free medical books  from the Internet? |  |  |  |  |
| 27. Search the Internet to find how a  particular clinical procedure is  carried out?. |  |  |  |  |
| 28. Searched the internet for the most current diagnostic test or therapy for a disease condition? |  |  |  |  |
| 29. Retrieve and download full-text  articles from an On-line / electronic journal e.g. (BMJ), through HINARI?  30. Find the most current available evidence to answer a clinical question  relating to your patient’s condition.  31. Find information on the diagnosis, prognosis, and treatment of an ailment? |  |  |  |  |

Thank you.
